# Digital Health as a Mechanism to Reduce Neonatal Intensive Care Unit Admissions: Retrospective Cohort Study

**DOI:** 10.2196/56247

**Published:** 2024-10-16

**Authors:** Alison K Brinson, Hannah R Jahnke, Natalie Henrich, Christa Moss, Neel Shah

**Affiliations:** 1 Maven Clinic, New York, NY, United States; 2 Department of Anthropology, University of North Carolina at Chapel Hill, Chapel Hill, NC, United States; 3 Carolina Population Center, University of North Carolina at Chapel Hill, Chapel Hill, NC, United States; 4 Department of Obstetrics and Gynecology, Beth Israel Deaconess Medical Center, Boston, MA, United States; 5 Harvard Medical School, Boston, MA, United States

**Keywords:** digital health, education, gestational conditions, Maven Clinic, mental health management, neonatal, NICU admissions, neonatal intensive care unit, mobile phone

## Abstract

**Background:**

Admission to the neonatal intensive care unit (NICU) is costly and has been associated with financial and emotional stress among families. Digital health may be well equipped to impact modifiable health factors that contribute to NICU admission rates.

**Objective:**

The aim of the study is to investigate how the use of a comprehensive prenatal digital health platform is associated with gestational age at birth and mechanisms to reduce the risk of admission to the NICU.

**Methods:**

Data were extracted from 3326 users who enrolled in a comprehensive digital health platform between January 2020 and May 2022. Multivariable linear and logistic regression models were used to estimate the associations between hours of digital health use and (1) gestational age at birth and (2) mechanisms to reduce the risk of a NICU admission. Multivariable logistic regression models estimated the associations between (1) gestational age at birth and (2) mechanisms to reduce the risk of a NICU admission and the likelihood of a NICU admission. All analyses were stratified by the presence of any gestational conditions during pregnancy.

**Results:**

For users both with and without gestational conditions, hours of digital health use were positively associated with gestational age at birth (in weeks; with gestational conditions: β=.01; 95% CI 0.0006-0.02; *P*=.04 and without gestational conditions: β=.01; 95% CI 0.0006-0.02; *P*=.04) and mechanisms that have the potential to reduce risk of a NICU admission, including learning medically accurate information (with gestational conditions: adjusted odds ratio [AOR] 1.05, 95% CI 1.03-1.07; *P*<.001 and without gestational conditions: AOR 1.04, 95% CI 1.02-1.06; *P*<.001), mental health management (with gestational conditions: AOR 1.06, 95% CI 1.04-1.08; *P*<.001 and without gestational conditions: AOR 1.03, 95% CI 1.02-1.05; *P*<.001), and understanding warning signs during pregnancy (with gestational conditions: AOR 1.08, 95% CI 1.06-1.11; *P*<.001 and without gestational conditions: AOR 1.09, 95% CI 1.07-1.11; *P*<.001). For users with and without gestational conditions, an increase in gestational age at birth was associated with a decreased likelihood of NICU admission (with gestational conditions: AOR 0.62, 95% CI 0.55-0.69; *P*<.001 and without gestational conditions: AOR 0.59, 95% CI 0.53-0.65; *P*<.001). Among users who developed gestational conditions, those who reported that the platform helped them understand warning signs during pregnancy had lower odds of a NICU admission (AOR 0.63, 95% CI 0.45-0.89; *P*=.01).

**Conclusions:**

Digital health use may aid in extending gestational age at birth and reduce the risk of NICU admission.

## Introduction

In the United States, most newborns who are born prematurely (before 37 weeks, 0 days gestation), with low birth weight (<2500 g), or with a health condition requiring special care are admitted to the neonatal intensive care unit (NICU) [1]. In 2022, approximately 9.5% of all infants born in the United States were admitted to the NICU [2]. NICUs are essential, as they improve infant survival and reduce morbidity for premature and sick infants [3]. However, admission to the NICU is costly and has been associated with financial and emotional stress among parents [4,5]. The parent-infant separation and parental stress resulting from NICU admission have been associated with poor maternal-infant attachment [6], lower breastfeeding rates [7], and higher rates of postpartum depression and anxiety [8], all of which may compromise subsequent pediatric health and development.

Several risk factors at the maternal level contribute to a higher likelihood of premature birth, which in turn increases the risk of NICU admission [9-11]. Primary maternal risk factors for a NICU admission include advanced age, chronic disease, substance use, preeclampsia, and peripartum infection [12-14]. Further, maternal stress, anxiety, and depression during pregnancy are associated with low birth weight and preterm birth [15-17]. At the institutional level, otherwise healthy infants who are born at hospitals with a high number of NICU beds have an increased likelihood of a NICU admission [10,18].

Previous interventions have successfully reduced the likelihood of a NICU admission with programs centered around increased patient education, access to care, and care coordination [19-21]. Digital health, including telemedicine and mobile apps, is increasingly used during pregnancy and may help address current gaps in prenatal care education by making pregnancy services more accessible and affordable [22,23]. By providing care coordination, continuous access to health care providers, and pregnancy-related educational materials, digital health may be well equipped to impact modifiable factors that contribute to NICU admission rates, including disease and mental health management.

In this study, we use data from Maven, a digital health platform, to explore how the use of a prenatal digital health platform that provides access to educational materials, telehealth through care providers, and care coordination is associated with gestational age at birth and mechanisms that may mitigate the risk of a NICU admission. First, we aimed to investigate the association between digital health use with gestational age at birth and mechanisms that could reduce the risk of a NICU admission (ie, mental health management and pregnancy education). Second, we explored whether gestational age at birth and the mechanisms to reduce risk are associated with a decreased likelihood of a NICU admission.

## Methods

### Ethical Considerations

All users consented to the use of their deidentified data for scientific research upon creating a Maven account. This study used deidentified data only, and the protocol was designated as exempt by WCG IRB (waiver 45 CFR § 46.104(d)(4)), an independent ethical review board.

### Study Setting and Design

**Figure 1.**
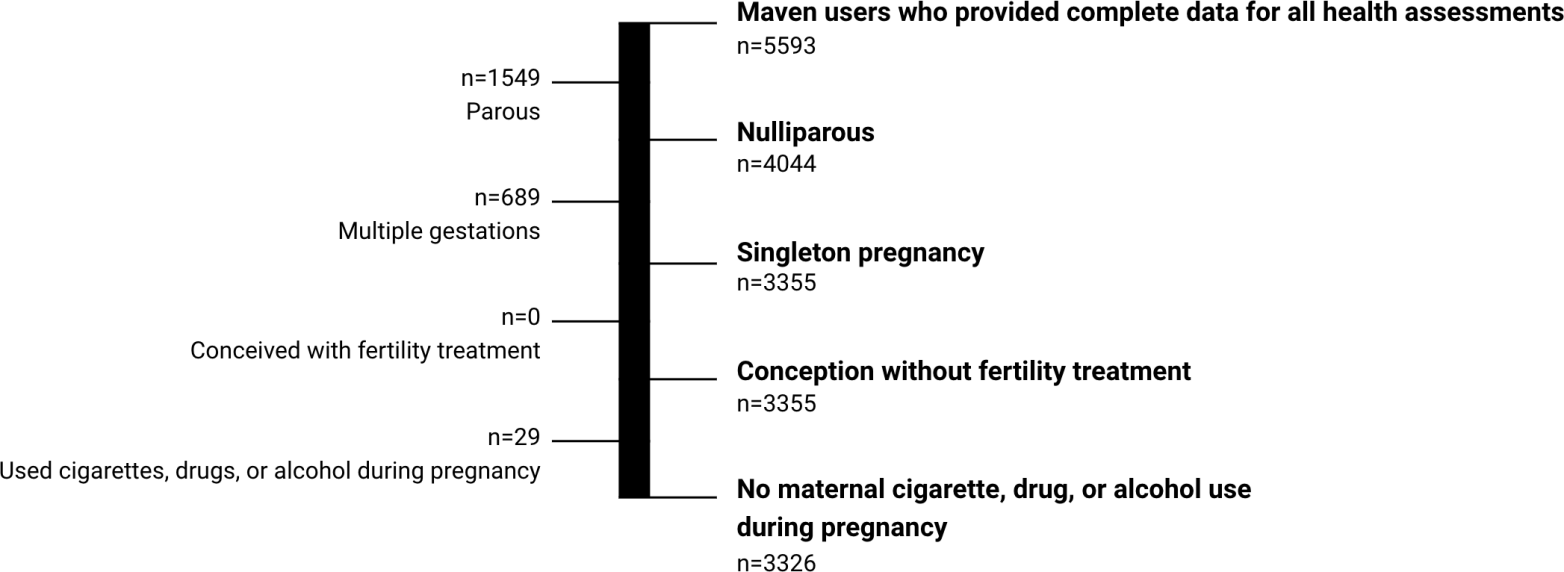
Flowchart of the users included in the current analysis.

This retrospective cohort study examined the associations between digital health use and NICU admission among pregnant individuals enrolled in Maven. Maven is a comprehensive digital health platform designed to support women’s and family’s health and complement routine prenatal care. Users receive free and unlimited access to Maven as an employer or health plan–sponsored benefit through their own or their partner’s employer. Maven provides a range of digital health education and support services within its platform. Users have access to a care advocate—an allied health professional such as a nurse or social worker—who serves as their primary point of contact within the platform. This advocate helps coordinate digital prenatal services and directs users to relevant providers and resources. Additionally, within the platform, users have access to articles, videos, live classes, and appointments with providers across a variety of specialties, including obstetrics and gynecology, mental health, nutrition, and others. This analysis used both platform-use data and user-reported data from the enrollment questionnaire (completed during pregnancy upon enrollment in Maven) and the postbirth questionnaire (completed after birth). Together, the enrollment and postbirth questionnaires collected data on user demographics, health characteristics, pregnancy outcomes, and the impact of the digital health platform during pregnancy. Data were extracted from 5593 Maven users in the United States who enrolled in Maven’s pregnancy program and completed both health questionnaires between January 1, 2020, and May 27, 2022 (Figure 1). We excluded users who had previously given birth (n=1549), had a multiple pregnancy (n=689), conceived with fertility treatment (n=0), or reported any cigarette, drug, or alcohol use during pregnancy (n=29). These exclusion decisions were made due to the strong associations between experiences in a previous pregnancy or birth [24], multiple gestation [25], and drug or alcohol use with NICU admission [26], independent of other factors.

### Outcome: NICU Admission

The primary study outcome was the birthing parent report of a NICU admission on the postbirth questionnaire. After birth, users were asked “Did you experience any of the following complications during delivery?” and could select all that apply from a list of complications. Any users who selected “My baby went to the NICU” were considered to have a NICU admission in this analysis.

### Hours of Maven Use

Hours of Maven use was the primary exposure in this analysis. The total number of active hours that each user spent on Maven was calculated from use data (automatically tracked within the platform) by summing the time spent with a care advocate, with a provider, messaging a provider, reading papers, attending web-based classes, or watching class recordings.

### Impacts of Digital Health Use

**Figure 2.**
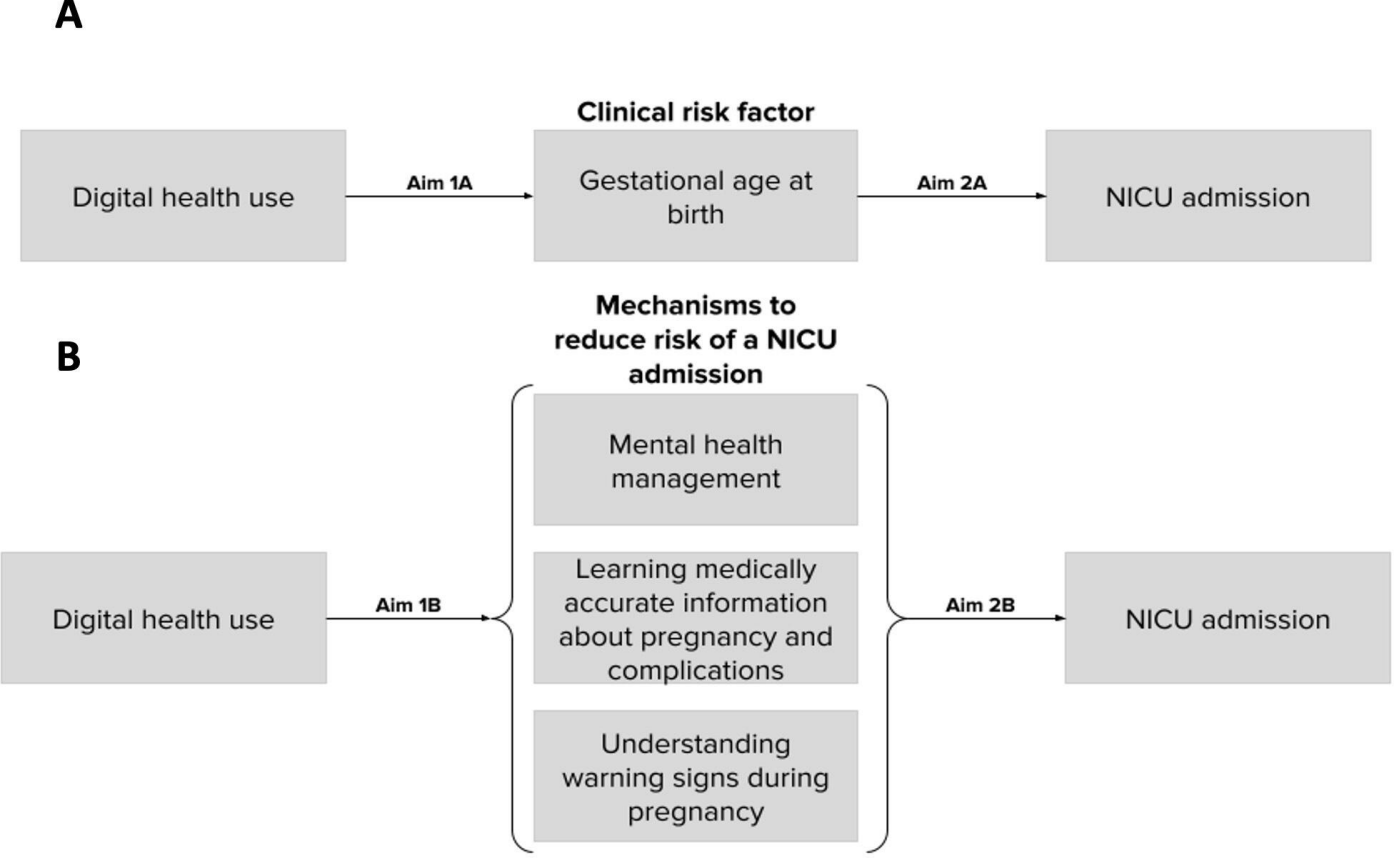
Conceptual model to explore the associations between digital health use and likelihood of an infant NICU admission. (A) Conceptual model to explore the associations between digital health use, gestational age at birth, and likelihood of an infant NICU admission. (B) Conceptual model to explore the associations between digital health use, mechanisms to reduce the risk of a NICU admission, and likelihood of an infant NICU admission. NICU: neonatal intensive care unit.

This analysis examined the association between time spent on the platform with gestational age at birth and 3 pathways by which digital health may reduce the risk of a NICU admission (Figure 2A and B). Gestational age at birth was a continuous variable calculated from the difference between the user-reported due date (collected on the enrollment questionnaire) and the user-reported baby date of birth (collected on the postbirth questionnaire).

The three mechanisms of NICU admission risk reduction were (1) mental health management, (2) learning medically accurate information about pregnancy and complications, and (3) understanding warning signs during pregnancy. Each of the mechanisms was assessed on the postbirth questionnaire. To assess mechanisms 1 and 2, users were asked “In what way did Maven influence your experience*?*” and could select all that apply from a list of ways Maven may have influenced their pregnancy, including “Maven helped me manage anxiety and/or depression” and “Maven helped me learn medically accurate information about pregnancy and complications.” If either of these options were selected, the item was coded as “yes,” and the items were coded as “no” if they were not selected. To assess mechanism 3, users were asked “Did Maven help you understand warning signs during pregnancy?” and users selected “yes” or “no.”

### Gestational Conditions

Given the strong associations between gestational conditions and infant health outcomes [27], all analyses were stratified by the presence of gestational conditions. To capture information on conditions that developed both before and after enrollment into Maven, the following conditions were assessed on both the enrollment and postbirth questionnaires: cholestasis, gestational diabetes, intrauterine growth restriction, high blood pressure, preeclampsia, eclampsia, vaginal blood loss (nonlabor related), problems with the placenta, issues with the cervix, excess or diminished amniotic fluid, infant large for gestational age, perinatal mood disorder, or hemolysis, elevated liver enzymes, and low platelet count syndrome. If a user reported the presence of any of the above conditions during their current pregnancy, they were coded as “Had at least one gestational condition” versus “No gestational conditions.”

### Covariates

Data from the enrollment questionnaire were used to create covariates. Ethnicity and race were categorized into “Hispanic or Latinx” and non-Hispanic or Latinx: “Asian or Pacific Islander,” “Black,” “White,” and “other” (comprised of users who reported their race as multiracial or American Indian. This category was created due to a small sample size of users who identified as multiracial or American Indian). To account for the societal and geographical factors that contribute to a NICU admission, we used the Centers for Disease Control and Prevention’s Social Vulnerability Index (SVI) [28]. SVI is a geographic measure of community vulnerability by using data from 4 domains: socioeconomic status, household composition and disability, minority status and language, and housing type and transportation. A continuous SVI score was assigned to each user based on their ZIP code, with an SVI closer to 1 representing high vulnerability. Chronic conditions were assessed as cumulative risk scores, calculated by adding the number of medical conditions reported by each user. Users reported their mode of birth (vaginal or cesarean) on the postbirth questionnaire.

### Statistical Methods

We conducted descriptive analyses to explore user demographic and medical characteristics stratified by the presence of gestational conditions. Chi-square or Fisher exact tests were used to assess categorical variables, and 2-tailed *t* tests and Wilcoxon rank-sum tests were used to assess continuous variables.

Adjusted linear regression was used to assess the association between time spent on the digital health platform and gestational age at birth (Figure 2; aim 1A). Adjusted logistic regression was used for all other aims. For aims 1A and 1B (Figure 2), assessing the associations between time spent using the digital health platform, gestational age at birth, and the 3 mechanisms to reduce the risk of NICU admission (mental health management, learning medically accurate information during pregnancy, and understanding warning signs during pregnancy), each component was assessed as an outcome in its own model. For aims 2A and 2B (Figure 2), examining whether gestational age at birth and mechanisms to reduce NICU admissions are associated with the likelihood of a NICU admission, each of the 4 components was assessed as the primary exposure in its own model with a report of a NICU admission as the outcome. Adjusted regression models controlled for age, race and ethnicity, mode of delivery, chronic conditions, SVI, and the number of days enrolled in Maven. All models estimated the effect with 95% CIs, and significance was determined when *P*<.05. All statistical analyses were performed using RStudio (Posit Software, PBC).

## Results

### Sample Characteristics

Our analytic sample consisted of 3326 pregnant individuals enrolled in the maternity program on the digital health platform. The mean age of our sample was 32.3 (SD 3.89) years. In total, 22.5% (747/3326) of users identified as non-Hispanic Asian or Pacific Islander, 46.5% (1547/3326) as non-Hispanic White, and 14.3% (475/3326) preferred not to disclose their race and ethnicity status (Table 1). Relatively few users reported the presence of any chronic medical conditions, with thyroid disease being the most prevalent (248/3326, 7.5%). During pregnancy, high blood pressure (482/3326, 14.5%) and gestational diabetes (357/3326, 10.7%) were the most common gestational conditions reported. The mean infant gestational age at birth was 39.3 (SD 1.57) weeks. A total of 68.9% (1799/3326) of users reported the digital health platform helped them learn medically accurate information about pregnancy and complications, 59.7% (1912/3326) reported the platform helped them understand warning signs during pregnancy, and 13.8% (361/3326) reported the platform helped them manage their mental health. A majority of 70.8% (2354/3326) of users reported having a vaginal birth, and 10.6% (353/3326) reported their infant was admitted to the NICU.

Compared to users who did not develop any gestational conditions during their pregnancy, users who developed one1 or more gestational conditions were less likely to be non-Hispanic White (44.1% [nn=613/1390], 44.1% vs. 48.2%[nn=934/1936], 48.2%) and more likely to be older (32.8 years y vs. 32.0 years); obese (21.3% [nn=296/1390], 21.3% vs. 9.4%[nn=182/1936], 9.4%); and have a a history of type 1 or type 2 diabetes (2.2% [nn=30/1390], 2.2% vs. 0.2%[nn=3/1936], 0.2%), hypertension (5.5% [nn=77/1390], 5.5% vs. 0.2% nn=3/1936], 0.2%), thyroid disease (8.9% [nn=124/1390, ]8.9% vs. 6.4% [nn=124/1936], 6.4%), anxiety (27.3% [nn= =379/1390], 27.3% vs. 18.5% [nn=359/1936], 18.5%), and depression (15.5% [nn=216/1390], 15.5% vs. 9.1% [nn=177/1936], 9.1%). Users with gestational conditions were more likely to report that the digital health platform helped them manage their mental health (15.9% [nn=177/1390], 15.9% vs. 12.3% [nn=184/1936, 12.3%]), deliver preterm (8.7% [nn=121/1390], 8.7% vs. 3.4% [nn=65/1936], 3.4%), have a baby who was admitted to the NICU (15.6% [nn=217/1390], 15.6% vs. 7.0% [nn=136/1936], 7.0%), and less likely to have a vaginal birth (62.7% [nn=872/1390], 62.7% vs. 76.5% [nn=1482/1936], 76.5%). The average amount of digital health use during pregnancy was 8.1 (SD 7.9) hours. Digital health use did not vary by the presence of gestational conditions.

**Table 1.** User demographic characteristics and select chronic conditions across gestational conditions groups^a^.

			Whole sample (N=3326)	0 gestational conditions (n=1936)	≥1 gestational conditions (n=1390)	*P* value
**User characteristics**						
	**Age, mean (SD)**		32.3 (3.89)	32.0 (3.63)	32.8 (4.18)	<.001
	**Race and ethnicity, n (%)**					.03
		Hispanic	325 (9.8)	179 (9.2)	146 (10.5)	
		Non-Hispanic Asian	747 (22.5)	419 (21.6)	328 (23.6)	
		Non-Hispanic Black	147 (4.4)	71 (3.7)	76 (5.5)	
		Non-Hispanic multiracial or American Indian	85 (2.6)	49 (2.5)	36 (2.6)	
		Non-Hispanic White	1547 (46.5)	934 (48.2)	613 (44.1)	
		Prefer not to say	475 (14.3)	284 (14.7)	191 (13.7)	
	**Social Vulnerability Index, mean (SD)**		0.38 (0.20)	0.38 (0.20)	0.38 (0.20)	.25
	**BMI (kg/m** ^**2**^ **), n (%)**					<.001
		Underweight (<18.5)	139 (4.2)	92 (4.8)	47 (3.4)	
		Normal weight (18.5‐24.9)	1906 (57.3)	1229 (63.5)	677 (48.7)	
		Overweight (25.0‐29.9)	803 (24.1)	433 (22.4)	370 (26.6)	
		Obese (≥30)	478 (14.4)	182 (9.4)	296 (21.3)	
**History of chronic conditions, n (%)**						
	Thyroid disease		248 (7.5)	124 (6.4)	124 (8.9)	.01
	Autoimmune disease		99 (3)	49 (2.5)	50 (3.6)	.07
	Hypertension		80 (2.4)	3 (0.2)	77 (5.5)	<.001
	Diabetes (type 1 or type 2)		33 (1)	3 (0.2)	30 (2.2)	<.001
	Blood disorder		24 (0.7)	10 (0.5)	14 (1)	.10
	Heart disease		19 (0.6)	8 (0.4)	11 (0.8)	.15
	Thrombophilia		16 (0.5)	6 (0.3)	10 (0.7)	.09
	Kidney disease		11 (0.3)	7 (0.4)	4 (0.3)	.77
	HIV/AIDS		1 (0)	0 (0)	1 (0.1)	.42
**History of reproductive conditions, n (%)**						
	Abnormal pap		322 (9.7)	176 (9.1)	146 (10.5)	.17
	Polycystic ovarian syndrome		223 (6.7)	117 (6)	106 (7.6)	.07
	Sexually transmitted disease		104 (3.1)	55 (2.8)	49 (3.5)	.26
	Endometriosis		74 (2.2)	36 (1.9)	38 (2.7)	.09
**History of mental health conditions, n (%)**						<.001
	Anxiety		738 (22.2)	359 (18.5)	379 (27.3)	
	Depression		393 (11.8)	177 (9.1)	216 (15.5)	
**Gestational conditions, n (%)**						—^b^
	High blood pressure		482 (14.5)	—	482 (34.7)	
	Gestational diabetes		357 (10.7)	—	357 (25.7)	
	Problems with the placenta		217 (6.5)	—	217 (15.6)	
	Preeclampsia, eclampsia, or HELLP^c^		212 (6.4)	—	212 (15.3)	
	Vaginal blood loss (excluding labor)		198 (6)	—	198 (14.2)	
	Infant large for gestational age		145 (4.4)	—	145 (10.4)	
	Excess or diminished amniotic fluid		134 (4)	—	134 (9.6)	
	Issues with the cervix		100 (3)	—	100 (7.2)	
	Intrauterine growth restriction		90 (2.7)	—	90 (6.5)	
	Perinatal mood disorder		70 (2.1)	—	70 (5)	
	Cholestasis		56 (1.7)	—	56 (4)	
**Pregnancy outcomes and complications**						
	Gestational age at birth (weeks), mean (SD)		39.3 (1.57)	39.6 (1.25)	38.9 (1.86)	<.001
	**Gestational age at birth category (weeks), n (%)**					<.001
		Preterm (<37)	186 (5.6)	65 (3.4)	121 (8.7)	
		Early term (37-<39)	717 (21.6)	334 (17.3)	383 (27.6)	
		Full term (39-<41)	2119 (63.7)	1320 (68.2)	799 (57.5)	
		Late term (41-<42)	295 (8.9)	211 (10.9)	84 (6)	
		Postterm (≥42)	9 (0.3)	6 (0.3)	3 (0.2)	
	NICU admission^d^, n (%)		353 (10.6)	136 (7)	217 (15.6)	<.001
	Vaginal birth, n (%)		2354 (70.8)	1482 (76.5)	872 (62.7)	<.001
**Mechanisms to reduce risk of a NICU admission, n (%)**						
	Mental health management		361 (13.8)	184 (12.3)	177 (15.9)	.01
	Learning medically accurate information about pregnancy and complications		1799 (68.9)	1018 (67.9)	781 (70.3)	.19
	Understanding warning signs during pregnancy		1912 (59.7)	1131 (60.7)	781 (58.2)	.15
**Digital health use**						
	Digital health use during pregnancy (hours), mean (SD)		8.10 (7.88)	8.02 (7.64)	8.21 (8.21)	.70

a Data are displayed for users who enrolled in Maven Clinic and gave birth between January 1, 2020, and September 19, 2022.

b Not applicable.

c HELLP: hemolysis, elevated liver enzymes, and low platelets.

d NICU: neonatal intensive care unit.

### Associations Between Digital Health Use, Gestational Age at Birth, and Mechanisms to Reduce Risk of a NICU Admission

In analyses of users who did not develop any gestational conditions during pregnancy, adjusted models revealed that for every 1-hour increase in digital health use, users experienced an increase in gestational age by 0.01 weeks (β=.01; 95% CI 0.0006-0.02; *P*=.04); a 9% increase in their odds of understanding warning signs during pregnancy (adjusted odds ratio [AOR] 1.09, 95% CI 1.07- 1.11; *P*<.001); a 3% increase in their odds of reporting that the platform helped the user manage their mental health (AOR 1.03, 95% CI 1.02-1.05; *P*<.001); and a 4% increase in their odds of reporting that the platform helped the user identify medically accurate information (AOR 1.04, 95% CI 1.02-1.06; *P*<.001; Table 2).

In analyses of users who developed 1 or more gestational conditions during pregnancy, adjusted models revealed that for every 1-hour increase in digital health use, users experienced an increase in gestational age by 0.01 weeks (β=.01; 95% CI 0.0006-0.02; *P*=.04), an 8% increase in their odds of understanding warning signs during pregnancy (AOR 1.08, 95% CI 1.06-1.11; *P*<.001), a 6% increase in their odds of reporting that the platform helped the user manage their mental health (AOR 1.06, 95% CI 1.04-1.08; *P*<.001), and a 5% increase in their odds of reporting that the platform helped the user identify medically accurate information (AOR 1.05, 95% CI 1.03-1.07; *P*<.001).

**Table 2.** Associations between hours of digital health use, gestational age at birth, and mechanisms to reduce risk of a neonatal intensive care unit admission^a^.

	No gestational conditions (n=1936)								≥1 gestational conditions (n=1390)							
	Gestational age at birth (weeks)		Understanding warning signs during pregnancy		Mental health management		Learning medically accurate information		Gestational age at birth (weeks)		Understanding warning signs during pregnancy		Mental health management		Learning medically accurate information	
	Adjusted β (95% CI)	*P* value	AOR^b^ (95% CI)	*P* value	AOR (95% CI)	*P* value	AOR (95% CI)	*P* value	Adjusted β (95% CI)	*P* value	AOR (95% CI)	*P* value	AOR (95% CI)	*P* value	AOR (95% CI)	*P* value
Digital health use (hours)	0.01(0.0006-0.02)	.04	1.09(1.07-1.11)	<.001	1.03(1.02-1.05)	<.001	1.04(1.02-1.06)	<.001	0.01(0.0006-0.02)	.04	1.08(1.06-1.11)	<.001	1.06(1.04-1.08)	<.001	1.05(1.03-1.07)	<.001

a Adjusted for age, Social Vulnerability Index, mode of delivery, race and ethnicity, chronic conditions, and days on Maven.

b AOR: adjusted odds ratio.

### Associations Between Gestational Age at Birth, Mechanisms to Reduce Risk of a NICU Admission, and the Likelihood of a NICU Admission

In analyses of users who did not develop any gestational conditions during pregnancy, adjusted logistic regression models found that for every 1-week increase in infant gestational age at birth, users experienced a 41% reduction in the odds of their infant being admitted to the NICU (AOR 0.59, 95% CI 0.53-0.65; *P*<.001; Table 3). Understanding warning signs during pregnancy (*P*=.05), learning medically accurate information about pregnancy and complications (*P*=.33), and mental health management (*P*=.83) were not significantly associated with the odds of a NICU admission at birth.

**Table 3.** Association between gestational age at birth, mechanisms to reduce risk of neonatal intensive care unit (NICU) admission, and the likelihood of a NICU admission^a^.

	0 gestational conditions (n=1936)			≥1 gestational conditions (n=1390)		
	Adjusted odds ratio of a NICU admission (95% CI)		*P* value	Adjusted odds ratio of a NICU admission (95% CI)		*P* value
Gestational age at birth (weeks)	0.59 (0.53-0.65)		<.001	0.62 (0.55-0.69)		<.001
Understanding warning signs during pregnancy	0.73 (0.53-1.01)		.05	0.63 (0.45-0.89)		.01
Learning medically accurate information	1.22 (0.83-1.83)		.33	0.92 (0.61-1.42)		.71
Mental health management	0.95 (0.56-1.52)		.83	1.45 (0.83-2.42)		.17

a Adjusted for age, Social Vulnerability Index, mode of delivery, race and ethnicity, chronic conditions, and days on Maven.

In analyses of users who developed 1 or more gestational conditions during pregnancy, adjusted logistic regression models found that for every 1-week increase in infant gestational age at birth, users experienced a 38% reduction in the odds of their infant being admitted to the NICU (AOR 0.62, 95% CI 0.55-0.69; *P*<.001; Table 3). Additionally, users who reported understanding warning signs during pregnancy experienced a 37% reduction in the odds of their infant being admitted to the NICU (AOR 0.63, 95% CI 0.45-0.89; *P*=.01; Table 3). Learning medically accurate information about pregnancy and complications (*P*=.71) and mental health management (*P*=.17) were not significantly associated with the odds of a NICU admission.

## Discussion

### Principal Results

The results of this retrospective cohort study suggest that the use of a comprehensive digital health platform during pregnancy helps users with and without gestational conditions extend their gestational age at birth, learn medically accurate information, manage their mental health, and identify warning signs during pregnancy. For all users, an increase in gestational age at birth was associated with a decreased likelihood of a NICU admission. Additionally, among users who developed 1 or more gestational conditions during pregnancy, those who reported that the digital health platform helped them identify warning signs during pregnancy had a 37% reduction in the odds of their infant being admitted to the NICU.

### Comparisons With Prior Work

Studies have found that birth outcomes can be improved through resources that support and complement traditional prenatal care. Previously, these influential resources have involved care coordination, transportation to appointments, and education [19-21]. Similarly, digital health can provide continuous care coordination and increased access to providers and educational materials [29]. This type of continuous support has been shown to have several benefits: improving in-clinic conversations with providers, influencing one’s mode of delivery and birth plan [30,31], managing mental health throughout pregnancy [32], and enabling users to identify and address problems immediately (vs waiting until their next in-person appointment) [33]. Additionally, digital health provides a patient-centered approach to complement routine prenatal care throughout pregnancy [34]. While routine prenatal care is limited by time constraints and other clinical needs of each visit, digital health is especially equipped to provide patient education and offer individualized content that may address patient concerns between in-person appointments [35]. In our study, using a digital health platform was linked to mental health management, learning medically accurate information, and understanding warning signs during pregnancy. Additionally, understanding these warning signs was associated with a reduced likelihood of NICU admission. While mental health management and learning medically accurate information about pregnancy were not associated with NICU admission, improvement in perinatal mental health and education may still be important in mitigating the risk of adverse obstetric outcomes including NICU, and these factors should continue to be investigated in future work.

In our sample, higher levels of digital health use were associated with an increase in gestational age at birth. It is important to note that while statistically significant, our effect size is quite small. For every 1 hour of digital health use, we saw an increase in gestational age by 0.01 weeks (approximately 2 hours). While the effect itself is small, identifying any signal is encouraging, given that an increase in gestational age decreases the risk of preterm birth and related complications, including a NICU admission [1]. This finding is consistent with previous research, which has shown that among pregnant individuals with gestational conditions, access to telehealth care is associated with a decreased likelihood of preterm birth [36,37]. Our results are especially promising because in this sample, digital health use was self-directed by the users. While the digital health platform offered resources and services aimed at reducing the risk of NICU admission, users were not given instructions on how frequently to use the platform and were not required to use specific types of care or resources. Future interventions with targeted protocols may yield even greater impacts.

Among users with 1 or more pregnancy-related conditions, users who reported that the digital health platform helped them understand warning signs during pregnancy had a reduced likelihood of a NICU admission. Given the rapid changes that occur as a pregnancy progresses, a diagnosis of a clinical condition like gestational hypertension or diabetes in an otherwise healthy individual may yield questions or concerns that arise outside of a monthly prenatal care appointment [34,38]. Digital health platforms can offer access to providers as well as educational content to help users manage their diagnosis outside of traditional in-person care [39,40].

### Limitations

Limitations of this study should be considered. First, our study population consisted primarily of commercially insured individuals who had internet access on a smartphone device or computer. Second, the majority identified as White and non-Hispanic, potentially limiting the generalizability and scalability of these findings.

### Conclusions

Digital health use may aid in reducing the risk of a NICU admission by extending gestational age at birth and helping individuals recognize warning signs during their pregnancy. As the use of digital health during pregnancy increases, this model of care may serve as a blueprint for how digital services may contribute to disease management during pregnancy and improve birth outcomes.

## Abbreviations

AOR: adjusted odds ratio
NICU: neonatal intensive care unit
SVI: Social Vulnerability Index
